# A randomized controlled trial evaluating the effect of two low-level laser irradiation protocols on the rate of canine retraction

**DOI:** 10.1038/s41598-022-14280-0

**Published:** 2022-06-16

**Authors:** Farah Y. Eid, Walid A. El-Kenany, Mohamed I. Mowafy, Ahmed R. El-Kalza, Myriam A. Guindi

**Affiliations:** 1 Department of Orthodontics, Faculty of Dentistry, Alexandria University, Champolion street, Azarita, Alexandria Egypt; 2 Department of Clinical Pathology, Faculty of Medicine, Alexandria University, Alexandria, Egypt

**Keywords:** Biomarkers, Medical research

## Abstract

The objective of this study was to evaluate the canine retraction rate with two low-level laser therapy (LLLT) irradiation protocols, involving both a high and a low application frequency. Twenty patients were randomly divided into two equal groups. In Group A, one side of the maxillary arch randomly received LLLT on days 0, 3, 7, 14, and every 2 weeks thereafter, whereas in Group B, one side received LLLT every 3 weeks. Tooth movement was checked every three weeks since the onset of canine retraction, over the 12-week study period. Moreover, Interleukin-1β (IL-1β) levels in the gingival crevicular fluid were assessed. Results revealed a significant increase in the canine retraction rate on the laser sides of groups A and B, in comparison with the control sides (*p* < 0.05), with no significant differences reported between the laser sides in both groups (*p* = 0.08–0.55). Also, IL-1β levels were significantly higher on the laser sides of both groups, in comparison with the control sides (*p* < 0.05). Therefore, LLLT can effectively accelerate tooth movement, with both frequent and less frequent applications, which is attributed to an enhanced biological response as reflected by the elevated IL-1β levels on the compression sides.

The prolonged orthodontic treatment time, which is usually around 20–30 months^1^, has been found to negatively affect patient compliance, in addition to posing several risks such as root resorption^2^, dental caries^3^, enamel decalcifications^3^, and periodontal problems^4,5^. Accordingly, several methods aiming to accelerate orthodontic tooth movement (OTM) have been proposed, including surgical and non-surgical adjuncts. Moreover, the effect of combining two acceleration techniques, as well as the impact of repeating the same acceleration procedure on the rate of OTM have both been investigated^6^.

Low-Level Laser Therapy (LLLT) has been one of the suggested non-surgical methods aiming to accelerate OTM, but contradictory results have been reported regarding its effectiveness in this field, with both positive^7,8^ and negative^9^ effects being documented. These conflicting results can be attributed to the difference in the laser application parameters used in each study, regarding the laser type, method of application, wavelength, irradiation dose, and exposure time, since these parameters have a direct correlation to the clinical results of laser application^10^.

Regarding the method of application, different laser irradiation protocols have been reported regarding the expedition of tooth movement. One of the commonly employed protocols involved laser application on days 0, 3, 7, 14, 21, and 30, with the same sequence being repeated every month, and this protocol has been adopted by several authors^11,12^. Others employed another protocol that is relatively close to the one previously stated and is also one of the common methods, where LLLT was applied on days 0, 3, 7, 14, and then every 15 days till the termination of the study period^13^. Additionally, a protocol that included a weekly application of low-level laser throughout the canine retraction period has been proposed^14^. However, a major drawback to those commonly adopted protocols was the high frequency of patient recall, which might not be convenient to everyone. Consequently, protocols requiring less patient recall have been adopted, such as those involving LLLT application on a monthly basis^8^, or every 3 weeks^15–18^.

Since orthodontic forces are known to induce bone remodeling, the occurrence of inflammatory changes is a prerequisite for this process to take place, and consequently result in tooth displacement^19^. According to several studies, one of the methods for the evaluation of the underlying biological events in the periodontal ligament, is through the assessment of the cytokines’ level in the gingival crevicular fluid (GCF)^20,21^. Interleukin-1β (IL-1β) is a substantially effectual cytokine in the process of bone metabolism^22^, and is considered one of the most powerful cytokines in the periodontium during the early stages of OTM^23^. Since a correlation between the level of IL-1β, and the survival, fusion, and activation of osteoclasts exists, therefore IL-1β can be considered a pertinent marker in calculating the extent of orthodontic tooth movement, in relation to the efficiency of alveolar bone remodeling^24^.

Therefore, the aim of our study was to evaluate and compare the effect of LLLT application with the commonly used protocol, involving a high application frequency on days 0, 3, 7, 14, and then every 2 weeks, versus its application every 3 weeks on the rate of canine retraction, in an attempt to reduce the frequency of patient recall. Additionally, the levels of IL-1β in the GCF have been assessed, with both protocols. The null hypothesis of the current study was that there is no difference regarding the rate of canine retraction with LLLT application, using both the tested protocols.

## Methods

### Study design

The study was a randomized controlled clinical trial, involving two parallel groups, each testing one of the LLLT application protocols. Each group adopted the split-mouth design, with one side serving as the control group, and the other side serving as the study group.

### Participants

Twenty female patients requiring the therapeutic extraction of maxillary first premolars, with subsequent canine retraction have been recruited for the sample, with an age range from 15 to 20 years. The sample size was calculated based on an alpha error of 5%, and an 80% study power. This calculation was based on the mean and standard deviation of canine retraction in the study by Doshi-Mehta and Bhad-Patil^7^, regarding LLLT application on days 0, 3, 7, 14, and then every 2 weeks (Group A), and those in the study by Qamruddin et al.^15^ regarding LLLT application every 3 weeks (Group B). Ethical approval was obtained from the Ethics Review Committee of the Faculty of Dentistry, Alexandria University, Alexandria, Egypt (IRB:00010556-IORG:0008839). Manuscript Ethics Committee number is 0111-01/2020. Approval was obtained on 21/01/2020. The trial was registered on ClinicalTrials.gov, with the name of the registry being “Two Low-level Laser Irradiation Protocols on the Rate of Canine Retraction.” The trial registration number is NCT04926389. The date of trial registration is 15/06/2021, and the URL is https://clinicaltrials.gov/ct2/show/NCT04926389. The trial started with patient recruitment on 02/05/2020, and it ended on 28/11/2021.

Patients were recruited from the outpatient clinic, Department of Orthodontics, Faculty of Dentistry, Alexandria University. Subjects were examined and screened, taking into consideration the following eligibility criteria: healthy systemic condition with no chronic diseases, no previous orthodontic treatment, adequate oral hygiene, and a healthy periodontium. A complete and thorough explanation regarding the study procedures was offered to both the participating patients and their parents, and accordingly, an informed consent has been obtained from each of enrolled subjects. All the research procedures were performed in accordance with the relevant guidelines and regulations as stated in the Declaration of Helsinki.

### Randomization and patient allocation

Before the commencement of canine retraction, the twenty recruited patients were randomly assigned to either Group A or Group B (10 per group), for low-level laser therapy application. Randomization was performed using a simple randomization process with an allocation ratio of 1:1. A box was prepared containing twenty folded pieces of paper, ten of which had the word “Group A” written on them, while the other ten papers had the word “Group B”. Each participant was asked to select one of the folded pieces of paper from the box and was accordingly assigned to one of the two groups. The same process was repeated once again within each group to assign one side of the maxillary arch to be the “study”, with the contralateral serving as the “control” in the split-mouth design.

### Patient preparation

The enrolled subjects were prepared for fixed orthodontic treatment by recording their medical and dental history, in addition to taking routine orthodontic records (intra-oral and extra-oral photographs, x-rays, and dental models). Patients were also asked to undergo full mouth scaling and polishing, followed by proper oral hygiene instructions (using the toothbrush, dental floss, and the interdental brush).

Maxillary and mandibular straight wire fixed Roth appliances with 0.022 $\times$ 0.028 inch slots were secured (Mini 2000; Ormco, USA) in all the recruited patients, where the bonding procedure was standardized in both groups, and was performed by the same operator. This was followed by patients’ referral for maxillary first premolars’ extraction, in order to provide sufficient time for healing of the extraction socket before the onset of canine retraction, which started approximately after 2 months from the extraction date. Leveling and alignment was then started and was considered complete when a 0.016 $\times$ 0.022 inch stainless steel arch wire could be placed passively in all the maxillary teeth.

Prior to the onset of canine retraction, the maxillary second premolars and first molars were ligated together, on both the experimental and control sides in the two groups, using a 0.009 inch wire in the form of figure of eight. Also, the maxillary incisors were ligated together in the same manner as the posterior segments, to aid in stabilization and to prevent their potential spacing.

Canine retraction in groups A and B, on both the experimental and control sides was performed using nickel-titanium (NiTi) closed-coil springs (Ormco, USA), stretched between the canine bracket hook and the hook on the molar tube, delivering a force of 150 g, as measured by a force gauge (Morelli, Brazil).

### Low-level laser application

**Figure 1:**
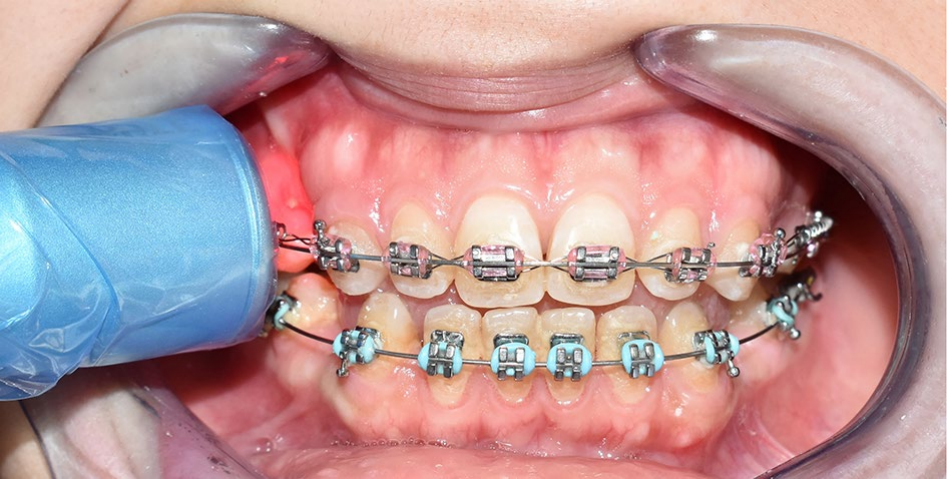
Optical fiber tip held against the maxillary canine root on the experimental side, at a distance of 1.5 cm, as per manufacturer instructions.

The low-level laser applied was a Diode laser (Wiser; Doctor Smile-Lambda Spa, Brendola, Italy), emitting infrared radiation at a wavelength of 980 nm, and an output power of 100 mW, in a continuous mode. The plane wave optical fiber (AB 2799; Doctor Smile-Lambda Spa, Brendola, Italy) dispensed a beam spot size of 1 cm^2^ using the flat top handpiece, and the irradiation was administered by positioning the optical fiber tip along the maxillary arch against the middle third of the canine root on the experimental side (1.5 cm as minimum on defocalization, as per manufacturer instructions), for 8 s (Fig. 1). The total energy density applied per episode was 8 J/cm^2^ (1 J/cm^2^ per second). The employed laser parameters are summarized in Table 1. Precautions were taken before laser application, where both the patient and the operator used protective eyeglasses supplied by the manufacturer, specific for the employed wavelength.

**Table 1:** Parameters of the employed low-level laser.

Parameter	Value
Wavelength	980 nm, continuous mode
Output power	100 mW
Beam size	1 cm^2^
Exposure time	8 s
Energy density	8 J/cm^2^ (1 J/cm^2^ per second)

In both groups, the split-mouth technique was employed, where each participant had one side of the maxillary arch randomly receiving LLLT, with the contralateral side serving as the control. In Group A, subjects received LLLT on days 0, 3, 7, 14, and every 2 weeks thereafter, whereas in Group B, LLLT was applied every 3 weeks on the experimental sides, throughout the study period (12 weeks). The laser beam was also held passively on the control sides of both groups, providing a placebo effect, as a part of the blinding process for the enrolled patients. Due to the nature of the intervention, the operator could not have been blinded at this stage.

### Collection of GCF samples

**Figure 2:**
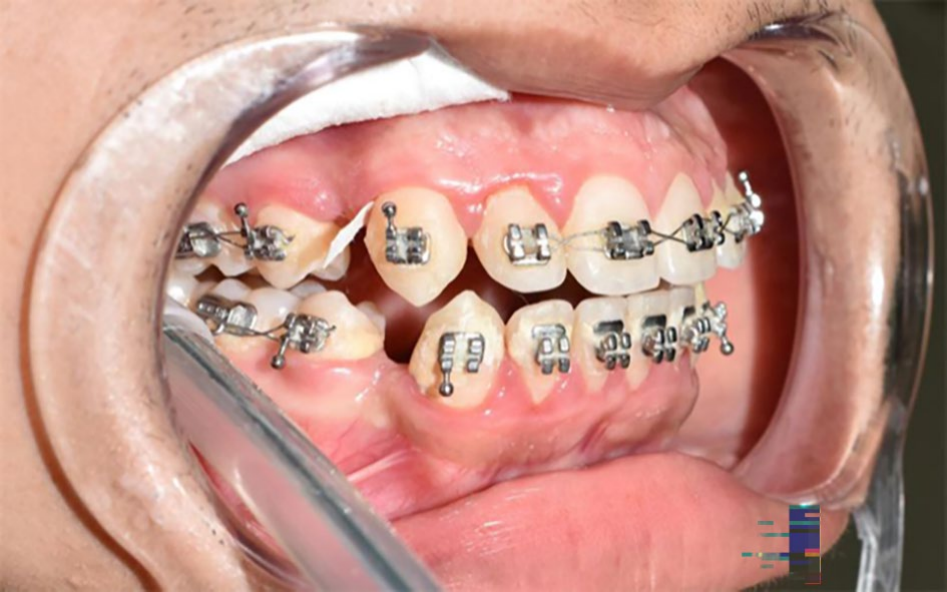
Collection of a GCF sample from the distal crevice of the maxillary canine.

Prior to sample collection, the maxillary canines on both sides were cleaned with a cotton pellet, isolated using a self-retaining retractor, suction, and cotton rolls, then gently air-dried for 5 s. Sample collection from the distal crevices of the maxillary canines was done using standardized filter-paper strips (Whatman, Maidstone, UK) that were cut into a standardized size of 2 × 10 mm^2^. Each paper strip was carefully inserted into the gingival crevice until mild resistance was felt, and it was left in situ for 60 s while maintaining proper isolation (Fig. 2). After removal, new strips were placed at 1-min intervals to obtain 4 strips at each site. Care was also taken to avoid any mechanical injury to the gingival crevices. Samples contaminated with saliva or blood were discarded, and new samples were collected. GCF samples were collected at baseline (before the onset of canine retraction), in addition to days 7, 14, and 21, from the distal crevices of the canines, in groups A and B, on both the experimental and control sides.

### Measurement of canine retraction

Alginate impressions (Ca37; Cavex, Haarlem, The Netherlands) were taken before starting canine retraction, and repeated every 3 weeks with each follow-up visit, throughout the 12-week study period. At each appointment, arch wires and coil springs were removed, and alginate impressions were taken, and subsequently poured with dental stone. Dental models were then trimmed and labeled with the patient’s name, number, and date. Afterwards, the stone models were scanned (inEos X5 CAD/CAM lab scanner; Dentsply Sirona, PA, USA), producing three-dimensional (3D) digital images of the dental models. The required measurements were performed using AutoCAD version 2013 (AutoCAD; Autodesk, USA). The clinician was blinded to the experimental and control sides during measurement to avoid unwarranted bias, and intra-examiner reliability was performed, where the measurements were repeated by the same operator one week later to check if there were measurement errors. Measurement error was calculated to be 6%.

**Figure 3:**
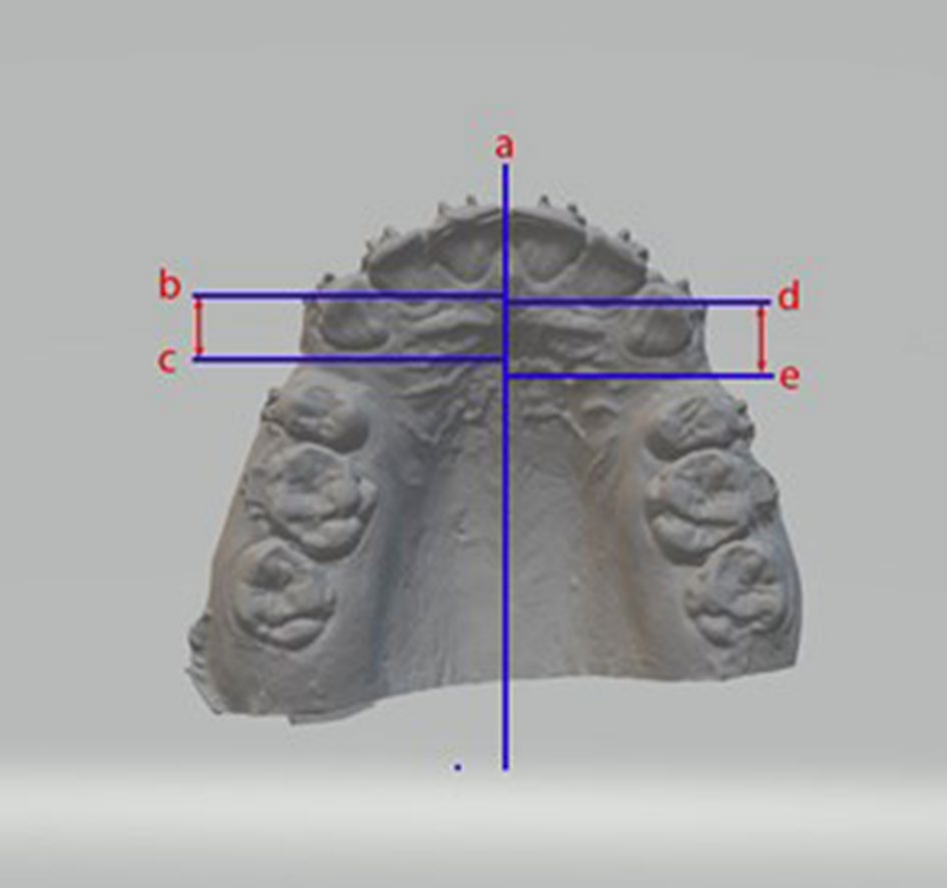
Landmarks were identified on the scanned image of the dental model for the measurement of canine retraction. (**a**). Mid-palatal raphe. (**b**, **d**). Cusp tips of the right and left maxillary canines, respectively. (**c**, **e**). Lines corresponding to the medial ends of the third right and left rugae, respectively.

**Figure 4:**
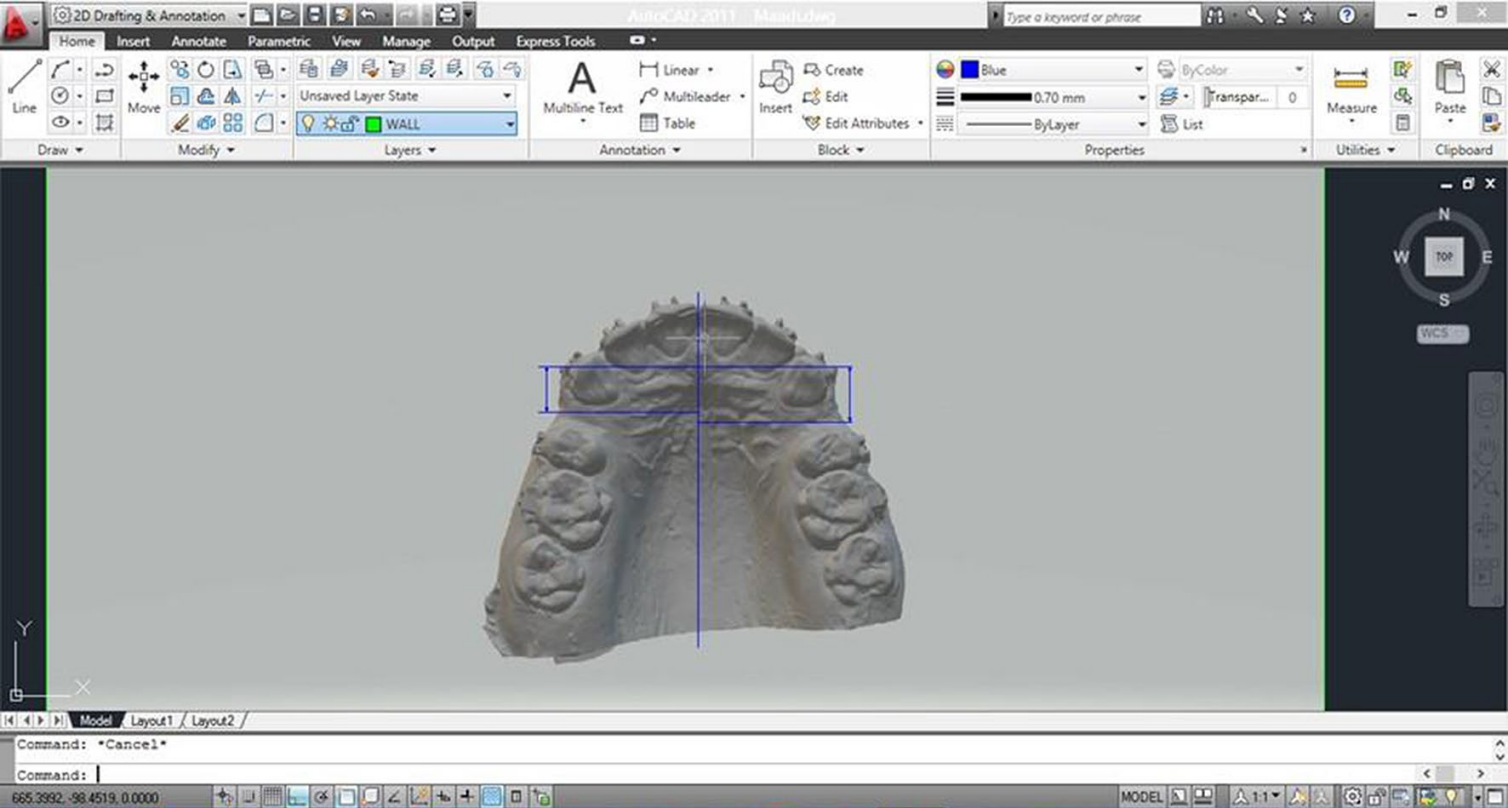
Measurement of canine retraction using AutoCAD.

Several landmarks were identified on the dental cast, including the mid-palatal raphe, the most medial points on the third right and left rugae, and the cusp tips of the right and left maxillary canines. Perpendicular lines were drawn from the medial points of the right and left third rugae, and the cusp tips of the right and left maxillary canines, to the mid-palatal raphe. The antero-posterior measurements were carried out between the canine lines and the third rugae lines bilaterally, to assess the rate of canine retraction (Figs. 3, 4).

### Determination of IL-1β levels in the GCF

**Figure 5:**
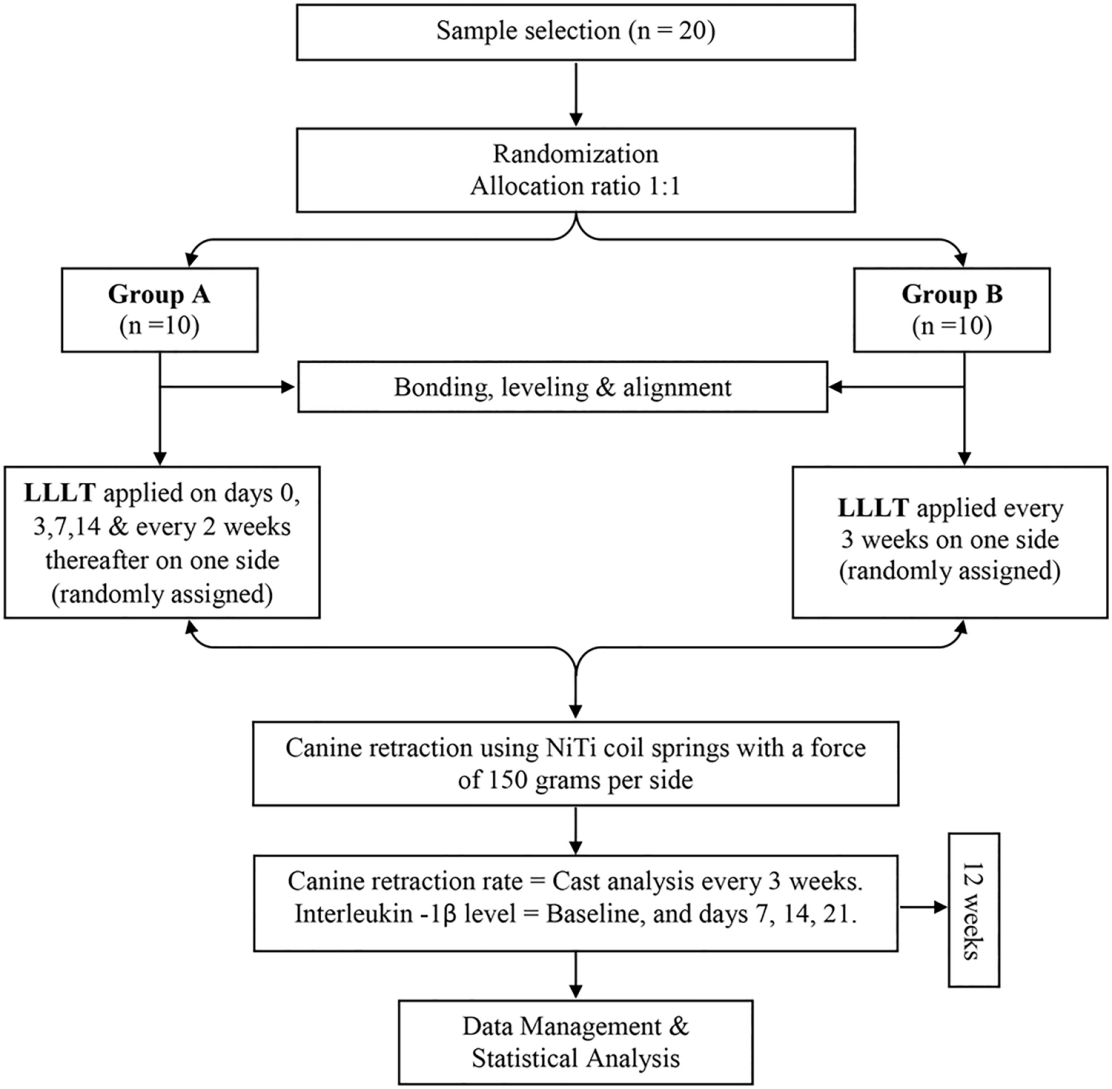
Research design flowchart.

Following their removal from the gingival crevice, each set of four filter paper strips collected from the same site was placed into an Eppendorf tube (Capp, Denmark), containing 100 µl of phosphate-buffered saline. The Eppendorf tubes were sealed and labeled, and the samples were immediately centrifuged using a centrifuge machine at 3000 rpm for 10 min (Hettich Universal 320R BC-HTX320; GMI, Min, USA), in order to recover the GCF samples from the paper strips. The Eppendorf tubes containing the samples, were then stored at − 20 °C until biochemical analysis. Analysis of IL-1β levels was performed using enzyme-linked immunosorbent assay (ELISA; Cloud-Clone, Hou, USA). The concentration of IL-1β was determined by comparing the optical density (OD) of the obtained samples to the standard curve, and accordingly, the linear regression equation of the standard curve was calculated. Results for IL-1β levels were finally reported in pg/ml/60 s^25^. A research design flowchart is represented in Fig. 5, summarizing the study procedures.

### Statistical analysis

Statistical analysis was performed using IBM SPSS for Windows version 23.0 (IBM; Armonk, NY, USA). All the quantitative variables showed normal distribution, so means, standard deviations (SD), and 95% confidence intervals (CI) were calculated, and parametric tests were used. Comparisons of quantitative variables (canine retraction, and IL-1β levels) between the two study groups were done using independent samples t-test, while comparisons between the laser and control sides in each group were done used paired t-test. Comparisons of canine retraction, and IL-1β levels at different time intervals within each group separately were done using repeated measures ANOVA, followed by multiple pairwise comparisons using Bonferroni adjusted significance levels. Significance was set at *p* value < 0.05.

## Results

**Figure 6:**
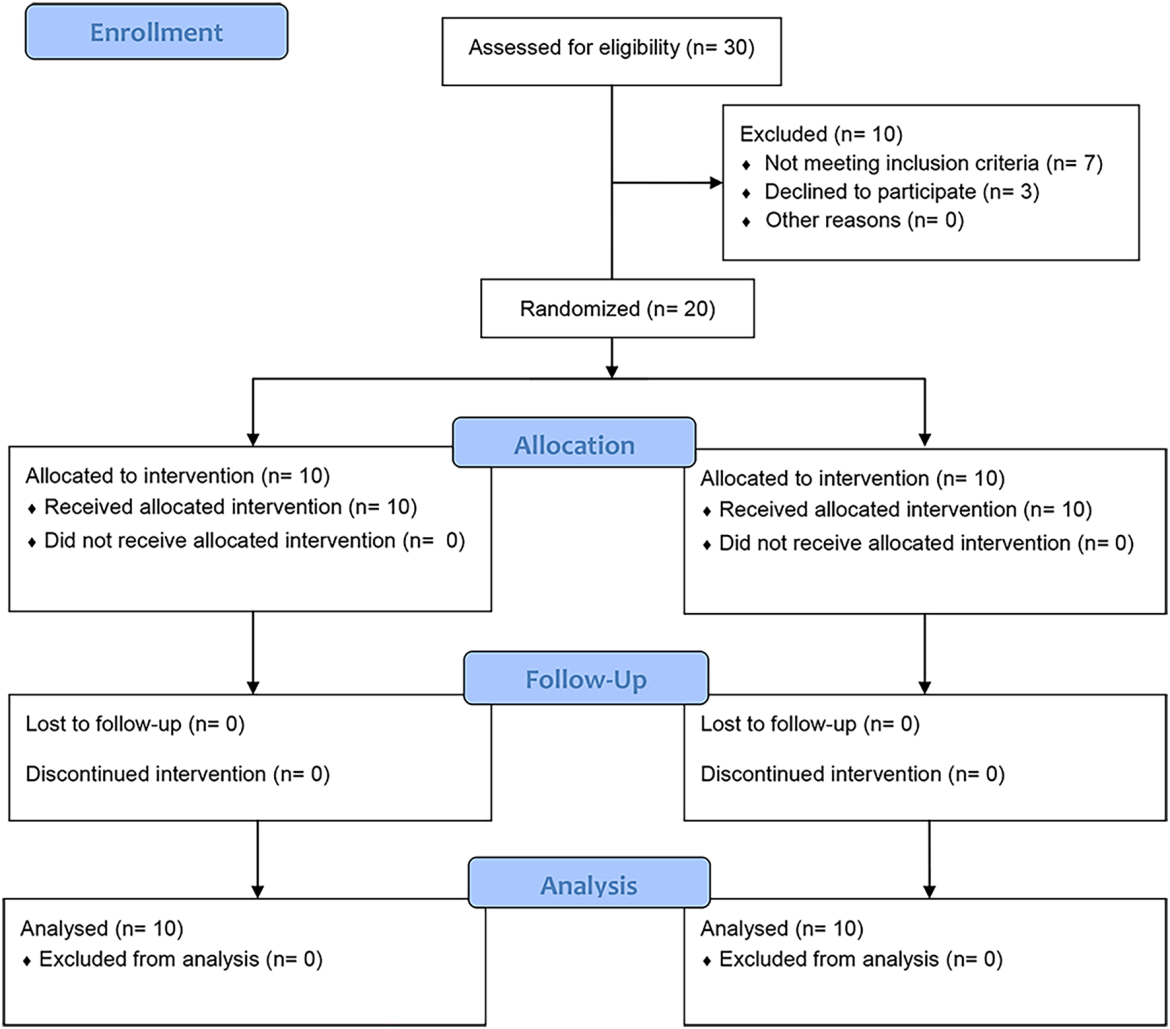
CONSORT Flow Diagram showing the patients’ flow throughout the clinical trial.

Over the course of the study, there were no subject dropouts in the pre-intervention period, nor throughout the rest of the study. All the twenty initially recruited subjects completed the entire 12-week study period (10 subjects per group). The patient flow throughout the trial is presented in Fig. 6, using a CONSORT Flow Diagram. Demographic data regarding the enrolled subjects in both groups A and B is presented in Table 2. No dropouts were recorded regarding the study models, which were obtained every three weeks for measurement of canine retraction. Also, all the obtained GCF samples were carefully handled, and analyzed.

**Table 2:** Demographic data regarding the enrolled study subjects in both groups A and B.

	Group A	Group B
Number of participants	10 subjects (n = 10)	10 subjects (n = 10)
Sex	Females	Females
Systemic condition	Healthy—No chronic diseases	Healthy—No chronic diseases
Previous orthodontic treatment	No previous orthodontic treatment	No previous orthodontic treatment
Periodontal condition	Healthy	Healthy

### Canine retraction rate

The amount of maxillary canine retraction at the different time points is described in Table 3, regarding both groups A and B. In Group A, the greatest mean distance (± SD) travelled by the maxillary canine has been reported at the 3^rd^ week to be 1.18 (± 0.04) mm on the laser side, and 0.85 (± 0.04) mm on the control side, with the difference between them being statistically significant (*p* < 0.001). However, the mean amount of tooth movement decreased at the 6th week on both the laser and control sides, then gradually increased afterwards over the 9th and 12th weeks, with the amount of tooth movement being significantly higher on the laser side in comparison with the control side (*p* < 0.001), at all the time points. The total amount of tooth movement (± SD) over the 12-week study period was significantly higher on the laser side with 4.45 (± 0.13) mm, compared to that on the control side which was 3.16 (± 0.14) mm (*p* < 0.001).

**Table 3:** Comparison of canine retraction (mm) between the laser and control sides at different time points, in the two study groups.

		Laser side	Control side	Difference	95% CI	Paired t *p* value
		Mean ± SD				
Group A	3 weeks	1.18 ± 0.04	0.85 ± 0.04	0.33 ± 0.03	0.31, 0.35	< 0.001*
	6 weeks	1.06 ± 0.05	0.74 ± 0.04	0.32 ± 0.05	0.28, 0.35	< 0.001*
	9 weeks	1.10 ± 0.03	0.78 ± 0.04	0.32 ± 0.04	0.29, 0.35	< 0.001*
	12 weeks	1.11 ± 0.05	0.79 ± 0.04	0.31 ± 0.06	0.26, 0.37	< 0.001*
	Total	4.45 ± 0.13	3.16 ± 0.14	1.29 ± 0.11	1.19, 1.38	< 0.001*
	RM-ANOVA p value	< 0.001*	< 0.001*			
Group B	3 weeks	1.14 ± 0.04	0.87 ± 0.03	0.27 ± 0.04	0.24, 0.30	< 0.001*
	6 weeks	1.05 ± 0.05	0.72 ± 0.02	0.33 ± 0.06	0.28, 0.38	< 0.001*
	9 weeks	1.08 ± 0.02	0.75 ± 0.02	0.33 ± 0.04	0.30, 0.36	< 0.001*
	12 weeks	1.08 ± 0.03	0.77 ± 0.02	0.31 ± 0.04	0.27, 0.34	< 0.001*
	Total	4.35 ± 0.12	3.10 ± 0.06	1.25 ± 0.13	1.13, 1.36	< 0.001*
	RM-ANOVA *p* value	< 0.001*	< 0.001*			

*SD* Standard deviation, *CI* Confidence interval, *RM-ANOVA* Repeated measures ANOVA.

*statistically significant at *p* value < 0.05.

In Group B, a similar pattern to that demonstrated in Group A has been followed, with significantly higher values of tooth movement being recorded on the laser side, in comparison to the control side at all time points (*p* < 0.001). At 3 weeks, the greatest tooth movement (± SD) was recorded, with a value of 1.14 (± 0.04) mm on the laser side, and 0.87 (± 0.03) mm on the control side. This was followed by a decrease in the tooth movement rate at the 6th week, and a gradual increase thereafter. The total amount of canine retraction (± SD) over the 12-week study period on the laser and control sides, was 4.35 (± 0.12) mm, and 3.10 (± 0.06) mm, respectively, and the difference between them was statistically significant (*p* < 0.001). In Table 4, a comparison between the laser and control sides within each of the study groups regarding the amount of canine retraction at different time points is depicted.

**Table 4:** Post-hoc pairwise comparisons of canine retraction at different time points, within the laser and control sides of each study group.

Group	Time point	Compared to	*P* value laser side	*P* value control side
Group A	3 weeks	6 weeks	< 0.001*	< 0.001*
		9 weeks	0.004*	0.001*
		12 weeks	0.08	0.004*
	6 weeks	9 weeks	0.02	0.054
		12 weeks	0.45	0.008*
	9 weeks	12 weeks	1.00	0.09
Group B	3 weeks	6 weeks	< 0.001*	< 0.001*
		9 weeks	0.008	< 0.001*
		12 weeks	0.002*	0.001*
	6 weeks	9 weeks	0.48	0.003*
		12 weeks	0.27	< 0.001*
	9 weeks	12 weeks	1.00	0.09

*Statistically significant using Bonferroni adjusted significance level.

#### Comparison between the laser sides in groups A and B regarding the amount of canine retraction

**Figure 7:**
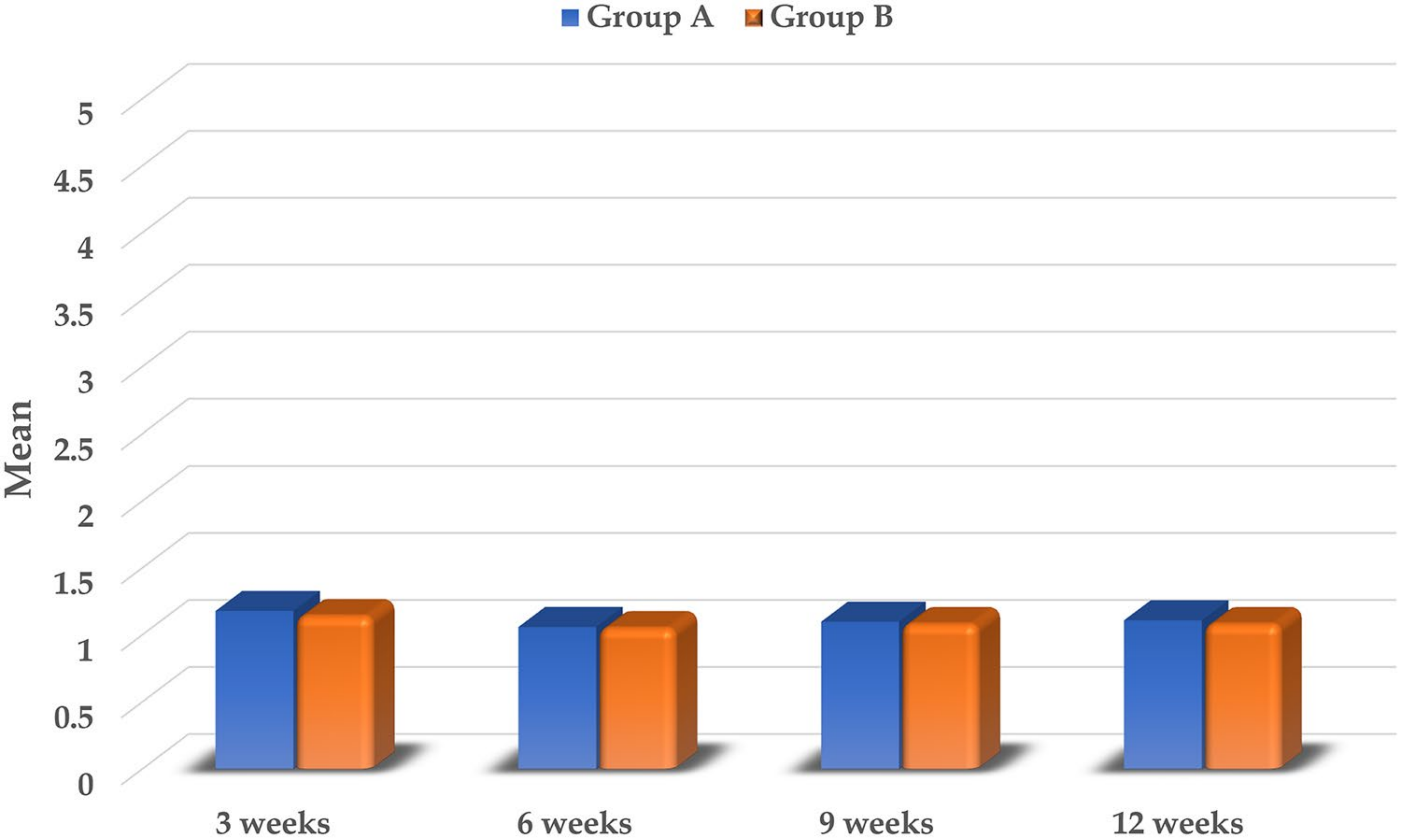
The amount of canine retraction (mm) on the laser sides in the two study groups at different time points, over the 12-week study period.

Although the amount of canine retraction at all the measured time points was higher on the laser sides in Group A, when compared to those in Group B, however, this difference was not considered statistically significant (*p* = 0.08–0.55). Regarding the percentage increase (± SD) in the amount of canine retraction accomplished by using each protocol, a 40.78 (± 4.81) % increase has been achieved with the protocol employed in Group A, while a 40.22 (± 4.80) % increase has been attained with the laser application protocol performed in Group B. However, although this percentage was slightly higher in Group A than in Group B, the difference between them was not statistically significant (*p* = 0.82). Moreover, the pattern of tooth movement was found to be relatively similar in both groups (Fig. 7).

### Interleukin-1β levels

The levels of IL-1β in both groups A and B, on the laser and control sides, at all the measured time points are depicted in Table 5. In Group A, insignificant differences have been noted between the laser and control sides at the baseline values of IL-1β (*p* = 0.56). The highest level of IL-1β (± SD) was recorded at day 7 on both the laser and control sides, with values of 0.152 (± 0.004) pg/ml/60 s, and 0.127 (± 0.004) pg/ml/60 s, respectively, and the difference between them was statistically significant (*p* < 0.001). A gradual decrease in IL-1β levels has been reported thereafter, on days 14 and 21, on both the laser and control sides, with the values on the laser side being significantly higher than those on the control side (*p* < 0.001).

**Table 5:** Comparison of IL-1β levels (pg/ml/60 s) between the laser and control sides at different time points, in the two study groups.

		Laser side	Control side	Difference	95% CI	Paired t *p* value
		Mean ± SD				
Group A	Baseline	0.089 ± 0.005	0.090 ± 0.005	− 0.001 ± 0.003	− 0.003, 0.002	0.56
	7 days	0.152 ± 0.004	0.127 ± 0.004	0.02 ± 0.004	0.02, 0.03	< 0.001*
	14 days	0.131 ± 0.002	0.111 ± 0.004	0.02 ± 0.004	0.01, 0.02	< 0.001*
	21 days	0.10 ± 0.004	0.09 ± 0.004	0.008 ± 0.004	0.005, 0.01	< 0.001*
	RM-ANOVA p value	< 0.001*	< 0.001*			
Group B	Baseline	0.089 ± 0.005	0.090 ± 0.005	− 0.001 ± 0.002	− 0.003, 0.001	0.20
	7 days	0.139 ± 0.004	0.122 ± 0.003	0.02 ± 0.004	0.01, 0.02	< 0.001*
	14 days	0.119 ± 0.001	0.110 ± 0.003	0.009 ± 0.002	0.007, 0.01	< 0.001*
	21 days	0.099 ± 0.001	0.092 ± 0.004	0.004 ± 0.002	0.002, 0.007	0.002*
	RM-ANOVA *p* value	< 0.001*	< 0.001*			

*SD* Standard deviation, *CI* Confidence interval, *RM-ANOVA* Repeated measures ANOVA.

*Statistically significant at *p* value < 0.05.

In Group B, a similar pattern to that of Group A regarding the IL-1β levels has been followed, with insignificant differences observed at the baseline between the laser and the control sides (*p* = 0.02). After 7 days, the peak of IL-1β level (± SD) was reached on both sides, with 0.139 (± 0.004) pg/ml/60 s on the laser side, and 0.122 (± 0.003) pg/ml/60 s on the control side, with the values on the laser side considered statistically higher (*p* < 0.001). This was followed by a gradual decrease in the IL-1β levels on days 14 and 21 on both sides, with significantly higher levels recorded on the laser side when compared to the control side at both time points (*p* = 0.001–0.002). A comparison between the laser and control sides within each of the study groups, regarding the levels of IL-1β at different time points is depicted in Table 6.

**Table 6:** Post-hoc pairwise comparisons of IL-1β levels at different time points, within the laser and control sides of each study group.

Group	Time point	Compared to	*P* value laser side	*P* value control side
Group A	Baseline	7 days	< 0.001*	< 0.001*
		14 days	< 0.001*	< 0.001*
		21 days	0.002*	0.04*
	7 days	14 days	< 0.001*	< 0.001*
		21 days	< 0.001*	< 0.001*
	14 days	21 days	< 0.001*	< 0.001*
Group B	Baseline	7 days	< 0.001*	< 0.001*
		14 days	< 0.001*	< 0.001*
		21 days	0.003*	0.02*
	7 days	14 days	< 0.001*	< 0.001*
		21 days	< 0.001*	< 0.001*
	14 days	21 days	< 0.001*	< 0.001*

*Statistically significant using Bonferroni adjusted significance level.

#### Comparison between the laser sides in groups A and B regarding the IL-1β levels

**Figure 8:**
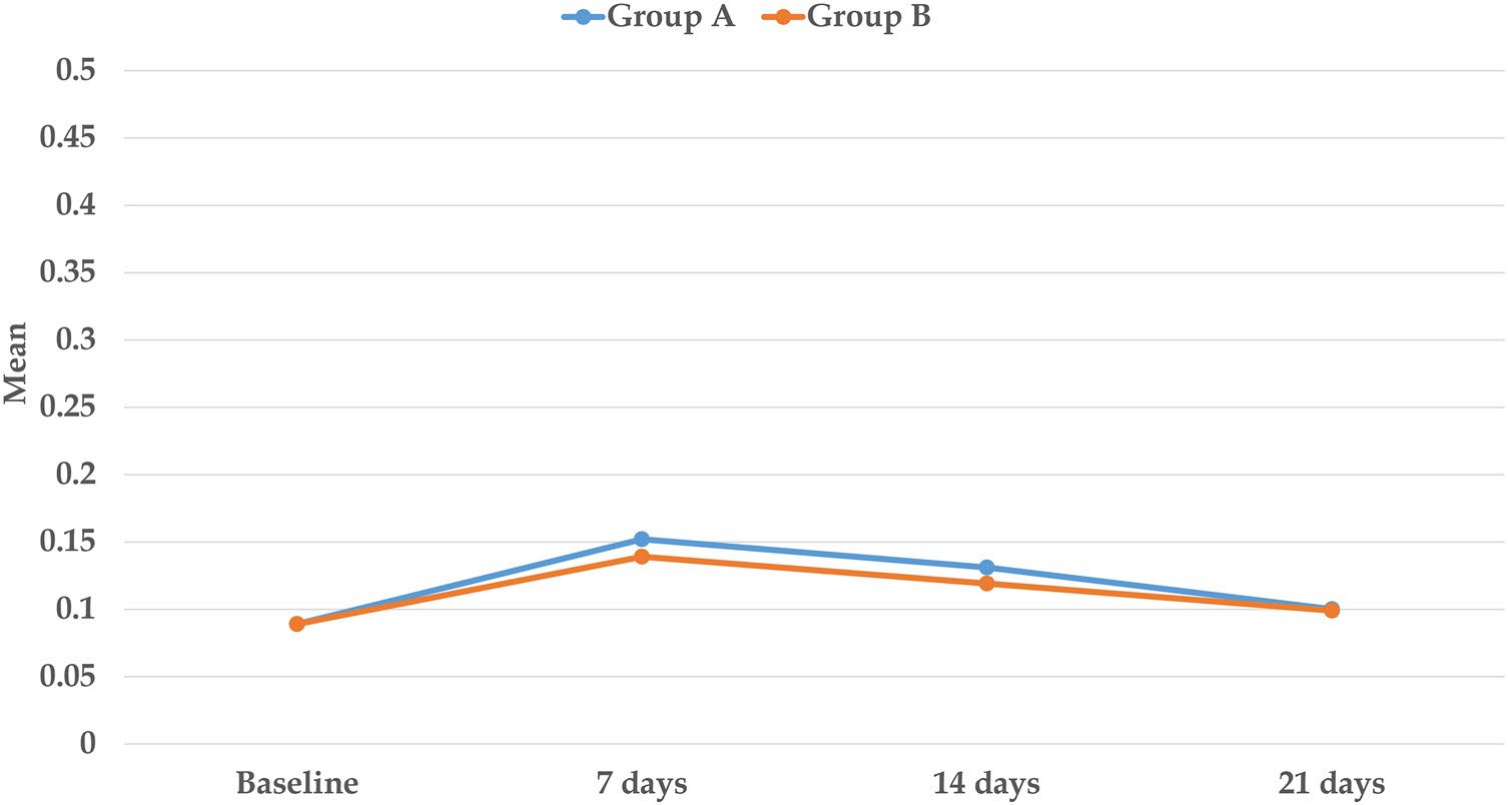
IL-1β levels (pg/ml/60 s) on the laser sides of the two study groups at different time points.

On comparing the levels of IL-1β between both study groups on the laser sides, insignificant differences have been documented at the baseline (*p* = 0.96). On the 7th and 14th days, statistically significant differences have been registered between the laser sides in both groups, with higher values belonging to the laser sides in Group A (*p* < 0.001). After 21 days, no significant differences have been documented between both groups (*p* = 0.26). The levels of IL-1β followed the same pattern in both groups, with a peak on day 7, and a gradual decrease over days 14 and 21 (Fig. 8).

## Discussion

The purpose of this study was primarily to evaluate and compare the effect of LLLT on the amount of canine retraction, using the protocol involving a high laser irradiation frequency, on days 0, 3, 7, 14, and every 2 weeks thereafter (Group A), versus the recently introduced protocol with less patient recall, where laser application has been performed at 3-week intervals (Group B). Both protocols have been documented in the literature, whether the common high frequency protocol^7,13,26^, or the 3-week application protocol^15,17,18^. According to the reported results of the current study, the null hypothesis was not rejected, where relatively similar amounts of canine movement have been achieved by implementing both the studied protocols.

The current study design was a clinical randomized controlled trial (RCT). RCTs are beheld as the gold standard for the assessment of intervention efficacy^27^. The split-mouth technique has also been employed, with its key advantage being the elimination of the inter-subject variability, where each patient acts as his/her own control, thus decreasing the number of participants required.

All the enrolled subjects required maxillary first premolars’ extraction, followed by canine retraction as a part of their orthodontic treatment. Since extractions can alter the rate of OTM through increasing the activity of inflammatory markers, which in turn could obscure the effect of LLLT, and provide a false indication regarding the levels of IL-1β with laser application, therefore extractions were undertaken before the commencement of treatment, providing ample time for healing of the extraction socket, and overcoming the effect of the regional acceleratory phenomenon^28^. This precaution also has been taken by some authors^11^, where the impact of LLLT on the OTM rate during canine retraction has been investigated, by measuring the levels of biological markers such as IL-1β, and Transforming growth factor beta 1 (TGF-β1) in the GCF.

The type of laser administered in this study was a Diode laser semiconductor, used at a wavelength of 980 nm as per manufacturer recommendation, in order to obtain the desirable bio-stimulatory effect. This can be explained by the fact that the longer the laser absorption wavelengths (650–1200 nm), the deeper the resultant tissue penetration^29^. However, this recommended wavelength has been employed in several other studies with both a positive acceleratory effect being yielded^8,30^, as well as a negative effect^14^.

Another principal element that influences the therapeutic and biostimulatory effect of LLLT is the dosage or the energy density. By reviewing the literature, a vast heterogeneity has been found in the administered energy dose of LLLT for the acceleration of OTM. Some authors used low energy densities ranging between 0.71^31^, 5^32,33^, 7.5^14^, and 8 J/cm^2^^34,35^, with positive results being reported, whereas other investigators also reported a positive acceleratory effect for LLLT on OTM rate at higher energy densities, such as 25 J/cm^2^^7,36^. In the present work, the administered low-level laser energy dose was 8 J/cm^2^, delivered through a single application of 8 s against the maxillary canine root, dispensing a beam spot size of 1 cm^2^ using the flat top handpiece. A direct correlation has been documented between the beam size and the laser penetration depth, which in turn justifies the use of the flat top handpiece in this study^29,37^. The same single application protocol with a large beam spot size was performed with leveling and alignment^8^, as well as with canine retraction^38^.

IL-1β is known to be a prominent pro-inflammatory cytokine at the start of OTM, and is considered a bone resorption marker. Accordingly, the levels of IL-1β have been assessed with laser application in several studies^11,39,40^, in an attempt to determine the correlation between them. In the current trial, the levels of IL-1β in the GCF have been assessed with the application of two different LLLT protocols on days 0, 7, 14, and 21, on the experimental and control sides in each group.

In the current study, the amount of canine retraction on the laser sides of both groups A and B was significantly higher than the control sides at all the assessed time points, with the peak reported at the 3rd week, followed by a decrease at the 6th week, then a gradual increase afterwards till the 12th week. The peak of canine movement that has been reported at the 3rd week can be explained by the effect of the initial displacement of the tooth, which includes: root displacement in the PDL, bone strain caused by bending and creep, and extrusion resulting from the inclined plane effect of the tooth pressing against the tapered alveolus^41^. Also, it has been found that all the active biological processes are accelerated while the bone is held in the deformed position^42^. The deceleration that has been observed afterwards between the 3rd and the 6th weeks, might be explained by the lag phase which could vary from 2 to 10 weeks, and it represents the period in which undermining resorption removes the bone adjacent to the crushed areas of the PDL, allowing the subsequent progression of tooth movement^43^. Another contributing factor to this observation could be the fact that the oxytalan fibers, collagen fibers, and alveolar bone remodeling on the tension side might also limit the rate of tooth displacement^44^. A similar tooth movement pattern has been found in a split-mouth study^45^ comparing between LLLT and corticotomy on the canine retraction rate, where they observed that the highest amount of tooth movement was noted in the 2nd and 5th weeks, followed by a sharp decrease at the 7th week on the laser side, but this finding was not reported on the corticotomy side.

A mean percentage increase in the moved distance by the maxillary canines has been reported to be 40.78% in Group A, and 40.22% in Group B, on the laser sides. This evident increase in tooth movement accompanying laser application could be explained on the cellular level by the absorption of laser energy by the photoreceptors in the electron transport respiratory chain, within the mitochondrial membranes. This action renders a short-term activation of the respiratory chain, resulting in oxidative phosphorylation, and an alteration in the redox status of both the cellular mitochondria and the cytoplasm. In turn, the promotive force of the cell increases through the enhanced supply of ATP. Also, an increase in the electrical potential of the mitochondrial membrane, cytoplasm alkalization, and nucleic acid synthesis takes place. Since ATP is known to be the energy currency for a cell, then LLLT promotes the normal functions of the cell, creating a favorable environment for tooth movement^46^. Hence, we can deduce from the obtained results that the application of LLLT as an adjunct to orthodontic treatment can successfully accelerate OTM, whether it was applied frequently like the protocol in Group A (on days 0, 3, 7, 14, and every 2 weeks thereafter), or if it was applied less frequently as carried out in Group B (every 3 weeks), and accordingly, the null hypothesis was not rejected.

The relatively equal acceleratory effect of the two tested LLLT application protocols reported in this study, might be due to the presence of a threshold for cellular activation, where enhanced cell activation through exposure to LLLT occurs initially^46^, but then the subsequently repeated exposures (as in Group A), do not result in further activation because of the saturated biological response. Thus, we can speculate that the effect of LLLT on the cellular level might not be cumulative. The concept of biological saturation has been previously described^47^ regarding the relation between the force level and the rate of tooth movement.

By reviewing the available literature, we compared the 1.4-fold (40–41%) increase in OTM yielded in our study using both laser protocols, with the results reported in several others. Comparable results have been reported in some studies^11,30,48,49^, while to the contrary, slightly lower acceleration values with LLLT application were registered in others^7,18,32,40^. On the other hand, much higher acceleration values than those reported in the current trial also have been reported, ranging from a 1.65-fold increase^17^, up to an almost twofold increase in OTM rate^15,34,39,50^, which could be attributed to the use of frictionless self-ligating brackets in some of them^15^. This divergence in the results reported in the literature is probably attributed to the different laser application protocols, wavelength, output power, irradiation time, energy density, treatment interval, and so on, making direct comparisons between different studies rather difficult. However, it has been stated that lower energy densities such as 2.5, 5, and 8 J/cm^2^, provided a better acceleration efficiency in comparison to higher energy densities^19^, and it is to be noted that the dosage applied in our trial was 8 J/cm^2^.

After analysis of the obtained GCF samples, the interpretation of IL-1β levels at the distal crevices (compression sides) showed a statistically significant increase from the baseline values at day 7, which was the peak, followed by a gradual decrease towards the baseline thereafter. This pattern was depicted in groups A and B, on both the laser and control sides. This can be explained by the fact that the initial stages of OTM are usually accompanied by an increased osteoclastic activity. IL-1β is also contemplated as the earliest detectable marker affiliated with bone resorption, and it has been reported that the expression of IL-1β is up-regulated following force application and diminished afterwards in a myriad of studies^11,20,51^.

Moreover, at all the measured time points aside from the baseline, higher levels of IL-1β were registered on the laser sides when compared with the control sides in both study groups, with a statistically significant difference between them. This finding suggests that low-level laser irradiation elicited an enhanced biologic response in the paradental tissues on the experimental sides, in the form of stimulation of osteoclast function on the compression sides during orthodontic tooth movement^11,52^. This effect that has been brought about by LLLT on the levels of IL-1β has been demonstrated in various studies^11,39,40^.

On comparing the IL-1β levels on the laser sides of both study groups, statistically higher levels have been documented in Group A in comparison with Group B, at the 7th and 14th days. This can be attributed to the greater number of exposures to laser irradiation in Group A over the 21-day observation period, where exposures were performed on days 0, 3, 7, and 14, whereas in Group B, only one exposure on day 0 was performed. However, although the levels of IL-1β were statistically higher on the laser sides in Group A, in comparison with the laser sides in Group B, this statistical difference was not reflected clinically in the amount of canine retraction, since there was no statistically significant difference recorded in the amount of canine retraction between the laser sides in both groups A and B, where in fact, equivalent amounts of canine movements have been achieved. Hence, we can say that statistical difference does not necessarily account for clinical significance.

## Conclusions

Low-level laser therapy when applied with the parameters employed in this study, can efficiently accelerate the rate of orthodontic tooth movement to approxiamtely 1.4-fold, whether applied with a high frequency, or with less frequent applications, coinciding with regular follow-ups, which might be more suitable to the patients.

The increase in the rate of orthodontic tooth movement with LLLT is accompanied by an increase in the levels of Interleukin-1β on the compression side, which suggests an enhanced bone remodeling process elicited with the administration of LLLT.

## Data Availability

The datasets used and/or analyzed during the current study are available from the corresponding author on reasonable request.

## Abbreviations

LLLT: Low-level laser therapy
IL-1β: Interleukin-1 beta
OTM: Orthodontic tooth movement
GCF: Gingival crevicular fluid
NiTi: Nickel-titanium
3D: Three-dimensional
ELISA: Enzyme-linked immunosorbent assay
OD: Optical density
SD: Standard deviation
CI: Confidence interval
RM-ANOVA: Repeated measures ANOVA
RCT: Randomized controlled trial
TGF-β1: Transforming growth factor beta 1

## References

[1] Skidmore KJ, Brook KJ, Thomson WM, Harding WJ (2006). Factors influencing treatment time in orthodontic patients. Am. J. Orthod. Dentofacial Orthop..

[2] Kurol J, Owman-Moll P, Lundgren D (1996). Time-related root resorption after application of a controlled continuous orthodontic force. Am. J. Orthod. Dentofacial Orthop..

[3] Årtun J, Thylstrup A (1986). Clinical and scanning electron microscopic study of surface changes of incipient caries lesions after debonding. Scand. J. Dent. Res..

[4] Ristic M, Svabic MV, Sasic M, Zelic O (2007). Clinical and microbiological effects of fixed orthodontic appliances on periodontal tissues in adolescents. Orthod. Craniofac. Res..

[5] Sanders NL (1999). Evidence-based care in orthodontics and periodontics: A review of the literature. J. Am. Dent. Assoc..

[6] Alfailany DT, Hajeer MY, Aljabban O, Mahaini L (2022). The effectiveness of repetition or multiplicity of different surgical and non-surgical procedures compared to a single procedure application in accelerating orthodontic tooth movement: A systematic review and meta-analysis. Cureus.

[7] Doshi-Mehta G, Bhad-Patil WA (2012). Efficacy of low-intensity laser therapy in reducing treatment time and orthodontic pain: A clinical investigation. Am. J. Orthod. Dentofacial Orthop..

[8] Caccianiga G (2017). Does low-level laser therapy enhance the efficiency of orthodontic dental alignment? Results from a randomized pilot study. Photomed. Laser Surg..

[9] Mistry D, Dalci O, Papageorgiou SN, Darendeliler MA, Papadopoulou AK (2020). The effects of a clinically feasible application of low-level laser therapy on the rate of orthodontic tooth movement: A triple-blind, split-mouth, randomized controlled trial. Am. J. Orthod. Dentofacial Orthop..

[10] Huang H, Williams RC, Kyrkanides S (2014). Accelerated orthodontic tooth movement: Molecular mechanisms. Am. J. Orthod. Dentofacial Orthop..

[11] Üretürk SE (2017). The effect of low-level laser therapy on tooth movement during canine distalization. Lasers Med. Sci..

[12] Heravi F, Moradi A, Ahrari F (2014). The effect of low level laser therapy on the rate of tooth movement and pain perception during canine retraction. Oral Health Dent. Manag..

[13] Isola G (2019). Effectiveness of low-level laser therapy during tooth movement: A randomized clinical trial. Materials.

[14] Yassaei S, Aghili H, Afshari JT, Bagherpour A, Eslami F (2016). Effects of diode laser (980 nm) on orthodontic tooth movement and interleukin 6 levels in gingival crevicular fluid in female subjects. Lasers Med. Sci..

[15] Qamruddin I (2017). Effects of low-level laser irradiation on the rate of orthodontic tooth movement and associated pain with self-ligating brackets. Am. J. Orthod. Dentofacial Orthop..

[16] Arumughan S (2018). A comparison of the rate of retraction with low-level laser therapy and conventional retraction technique. Contemp. Clin. Dent..

[17] Qamruddin I (2021). Photobiostimulatory effect of a single dose of low-level laser on orthodontic tooth movement and pain. Pain Res. Manag..

[18] Garg NJ (2014). Effect of 810 nm diode laser therapy on the rate of extraction space closure. J. Indian Orthod. Soc..

[19] Ge MK (2015). Efficacy of low-level laser therapy for accelerating tooth movement during orthodontic treatment: A systematic review and meta-analysis. Lasers Med. Sci..

[20] Uematsu S, Mogi M, Deguchi T (1996). Interleukin (IL)-1β, IL-6, tumor necrosis factor-α, epidermal growth factor, and β2-microglobulin levels are elevated in gingival crevicular fluid during human orthodontic tooth movement. J. Dent. Res..

[21] Başaran G, Özer T, Kaya FA, Hamamci O (2006). Interleukins 2, 6, and 8 levels in human gingival sulcus during orthodontic treatment. Am. J. Orthod. Dentofacial Orthop..

[22] Krishnan V, Davidovitch ZE (2006). Cellular, molecular, and tissue-level reactions to orthodontic force. Am. J. Orthod. Dentofacial Orthop..

[23] d'Apuzzo F (2013). Biomarkers of periodontal tissue remodeling during orthodontic tooth movement in mice and men: Overview and clinical relevance. Sci. World J..

[24] Teixeira CC (2010). Cytokine expression and accelerated tooth movement. J. Dent. Res..

[25] Wassall RR, Preshaw PM (2016). Clinical and technical considerations in the analysis of gingival crevicular fluid. Periodontol..

[26] AlSayed Hasan MMA, Sultan K, Hamadah O (2017). Low-level laser therapy effectiveness in accelerating orthodontic tooth movement: A randomized controlled clinical trial. Angle Orthod..

[27] Stang A (2011). Randomized controlled trials-an indispensible part of clinical research. Dtsch. Arztebl. Int..

[28] Frost HM (1983). The regional acceleratory phenomenon: A review. Henry Ford Hosp. Med. J..

[29] Farkas JP, Hoopman JE, Kenkel JM (2013). Five parameters you must understand to master control of your laser/light-based devices. Aesthet. Surg. J..

[30] Jivrajani SJ, Bhad WA (2020). Effect of Low Intensity Laser Therapy (LILT) on MMP-9 expression in gingival crevicular fluid and rate of orthodontic tooth movement in patients undergoing canine retraction: A randomized controlled trial. Int. Orthod..

[31] Genc G (2013). Effect of low-level laser therapy (LLLT) on orthodontic tooth movement. Lasers Med. Sci..

[32] Cruz DR, Kohara EK, Ribeiro MS, Wetter NU (2004). Effects of low-intensity laser therapy on the orthodontic movement velocity of human teeth: A preliminary study. Lasers Surg. Med..

[33] Kochar GD (2017). Effect of low-level laser therapy on orthodontic tooth movement. J. Indian Orthod. Soc..

[34] Youssef M (2008). The effect of low-level laser therapy during orthodontic movement: A preliminary study. Lasers Med. Sci..

[35] Hussein FA, Al-Haj AM, Shendy MAER, El-Awady AA (2020). Consequence of two protocols and energy doses of low-level laser therapy on the rate of orthodontic canine retraction: A prospective clinical evaluation. Egypt. Dent. J..

[36] Fernandes MR, Suzuki SS, Suzuki H, Martinez EF, Garcez AS (2019). Photobiomodulation increases intrusion tooth movement and modulates IL-6, IL-8 and IL-1β expression during orthodontically bone remodeling. J. Biophotonics.

[37] Ash C, Dubec M, Donne K, Bashford T (2017). Effect of wavelength and beam width on penetration in light-tissue interaction using computational methods. Lasers Med. Sci..

[38] Abd El-Ghafour M, El-Ashmawi NA, El-Beialy AR, Fayed MMS, Eid F (2017). Effect of low level laser therapy on the rate of canine retraction in orthodontic patients: A split-mouth randomized controlled trial. Orthod. Pract. US.

[39] Varella AM, Revankar AV, Patil AK (2018). Low-level laser therapy increases interleukin-1β in gingival crevicular fluid and enhances the rate of orthodontic tooth movement. Am. J. Orthod. Dentofacial Orthop..

[40] Zheng J, Yang K (2021). Clinical research: Low-level laser therapy in accelerating orthodontic tooth movement. BMC Oral Health.

[41] Roberts W, Garetto L, Katona T, Carlson D, Goldstein S (1992). Bone biodynamics in orthodontic and orthopedic treatment. Eur. J. Orthod..

[42] Baumrind S (1969). A reconsideration of the propriety of the “pressure-tension” hypothesis. Am. J. Orthod..

[43] Graber LW, Vanarsdall RL, Vig KW, Huang GJ (2016). Orthodontics-e-book: Current Principles and Techniques.

[44] Kohno T, Matsumoto Y, Kanno Z, Warita H, Soma K (2002). Experimental tooth movement under light orthodontic forces: Rates of tooth movement and changes of the periodontium. J. Orthod..

[45] El-Ashmawi NM (2018). Effect of surgical corticotomy versus low level laser therapy (LLLT) on the rate of canine retraction in orthodontic patients. Orthod. Pract. US.

[46] Reza F, Farzaneh A, Katayoun KA, Nikoo T (2011). Laser in Orthodontics.

[47] Alikhani M (2015). Saturation of the biological response to orthodontic forces and its effect on the rate of tooth movement. Orthod. Craniofac. Res..

[48] Lalnunpuii H, Batra P, Sharma K, Srivastava A, Raghavan S (2020). Comparison of rate of orthodontic tooth movement in adolescent patients undergoing treatment by first bicuspid extraction and en-mass retraction, associated with low level laser therapy in passive self-ligating and conventional brackets: A randomized controlled trial. Int. Orthod..

[49] Impellizzeri A (2020). Photobiomodulation therapy on orthodontic movement: Analysis of preliminary studies with a new protocol. Int. J. Environ. Res. Public Health.

[50] da Silva Sousa MV, Scanavini MA, Sannomiya EK, Velasco LG, Angelieri F (2011). Influence of low-level laser on the speed of orthodontic movement. Photomed. Laser Surg..

[51] Lee KJ, Park YC, Yu HS, Choi SH, Yoo YJ (2004). Effects of continuous and interrupted orthodontic force on interleukin-1β and prostaglandin E2 production in gingival crevicular fluid. Am. J. Orthod. Dentofacial Orthop..

[52] Kawasaki K, Shimizu N (2000). Effects of low-energy laser irradiation on bone remodeling during experimental tooth movement in rats. Lasers Surg. Med..

